# Sweet-Tasting Natural Proteins Brazzein and Monellin: Safe Sugar Substitutes for the Food Industry

**DOI:** 10.3390/foods12224065

**Published:** 2023-11-08

**Authors:** Tamara S. Novik, Elena I. Koveshnikova, Anatoly A. Kotlobay, Lyudmila P. Sycheva, Karine G. Kurochkina, Olga A. Averina, Maria V. Belopolskaya, Petr V. Sergiev, Olga A. Dontsova, Vassili N. Lazarev, Igor V. Maev, Margarita G. Kostyaeva, Artem V. Eremeev, Svetlana I. Chukina, Maria A. Lagarkova

**Affiliations:** 1 Scientific Research Centre Pharmbiomed, Selskohozjajstvennaja Str., 12a, Moscow 129226, Russia; novik.tamara@mail.ru (T.S.N.); koveshnikova.e.i@yandex.ru (E.I.K.); kar.kur.49@yandex.ru (K.G.K.); feruza7491@mail.ru (S.I.C.); 2 Lopukhin Federal Research and Clinical Center of Physical-Chemical Medicine, Malaya Pirogovskaya Str. 1a, Moscow 119435, Russia; an_kotlobay@rcpcm.org (A.A.K.); lazar0@mail.ru (V.N.L.); art-eremeev@yandex.ru (A.V.E.); 3 Burnasyan Federal Medical Biophysical Center of Federal Medical Biological Agency, Zhivopisnaya Str., 46, Moscow 123182, Russia; lpsycheva@mail.ru; 4 A.N. Belozersky Institute of Physico-Chemical Biology, Lomonosov Moscow State University, Leninskie Gory 1, Moscow 119991, Russia; averina.olga.msu@gmail.com (O.A.A.); petya@genebee.msu.ru (P.V.S.); olga.a.dontsova@gmail.com (O.A.D.); 5 Institute of Mitoengineering MSU, Leninskie Gory 1, Moscow 119991, Russia; belopolskaya@yandex.ru; 6 Department of Propaedeutics of Internal Diseases and Gastroenterology, Moscow State University of Medicine and Dentistry, Delegatskaya St. 20/1, Moscow 103473, Russia; igormaev@rambler.ru; 7 Faculty of Medicine, Peoples Friendship University of Russia Named after Patrice Lumumba, Miklukho-Maklaya Str.6, Moscow 117198, Russia; kostyaeva.71@mail.ru

**Keywords:** sweet proteins, natural sweeteners, food additives, safety, toxicology studies

## Abstract

This article presents the results of a comprehensive toxicity assessment of brazzein and monellin, yeast-produced recombinant sweet-tasting proteins. Excessive sugar consumption is one of the leading dietary and nutritional problems in the world, resulting in health complications such as obesity, high blood pressure, and cardiovascular disease. Although artificial small-molecule sweeteners widely replace sugar in food, their safety and long-term health effects remain debatable. Many sweet-tasting proteins, including thaumatin, miraculin, pentadin, curculin, mabinlin, brazzein, and monellin have been found in tropical plants. These proteins, such as brazzein and monellin, are thousands-fold sweeter than sucrose. Multiple reports have presented preparations of recombinant sweet-tasting proteins. A thorough and comprehensive assessment of their toxicity and safety is necessary to introduce and apply sweet-tasting proteins in the food industry. We experimentally assessed acute, subchronic, and chronic toxicity effects, as well as allergenic and mutagenic properties of recombinant brazzein and monellin. Our study was performed on three mammalian species (mice, rats, and guinea pigs). Assessment of animals’ physiological, biochemical, hematological, morphological, and behavioral indices allows us to assert that monellin and brazzein are safe and nontoxic for the mammalian organism, which opens vast opportunities for their application in the food industry as sugar alternatives.

## 1. Introduction

According to the World Health Organization, about 2 billion people in the world, including tens of millions of children, suffer from overweight and obesity [1]. One of the main reasons for the increasing number of overweight people is excessive sugar consumption, which leads to the development of chronic pathologies such as type 2 diabetes, hyperlipidemia, hypertension, ischemia, and other cardiovascular diseases [2,3], cognitive impairment, and depression [4]. From the 19th century, when an explosive increase in sugar production occurred [3,5], to the present day, human populations’ sugar consumption has been constantly increasing [3]. The main food sources of excess sugar in daily consumption are sweetened beverages and sweet baked goods [6,7]. Reducing the sugar content in sweet products is an urgent problem and a necessity for health in many countries of the world [8]. At the same time, it is desirable to preserve the taste and sweetness level of products. One feasible solution to this problem may be substituting sugar with natural sweet-tasting proteins isolated from several wild plants growing in tropical regions of Africa and Southeast Asia [9]. To date, eight natural sweet-tasting proteins have been isolated from plants and structurally and functionally studied: miraculin, monellin, brazzein, thaumatin, neoculin, mabinlin, curculin, and pentadin [10]. Their amino acid compositions and structures were characterized [11], their mechanisms of interaction with sweet taste receptor T1R2/T1R3 was described [12], and production methods suitable to obtain high amounts of the recombinant proteins, including mutant ones, were developed [13,14,15]. Brazzein and monellin are among the most investigated proteins in this group.

Monellin was isolated from the leaves and fruits of the African plant *Dioscoreophyllum cumminsii* [16]. Natural monellin is a polypeptide with a molecular weight of 11.4 kDa, consisting of 94 amino acids arranged in two non-covalently associated chains [17]. According to various estimates, monellin is up to 3000 times sweeter than sucrose [18]. The use of natural monellin as a sweetener is complicated by the fact that the compound is not temperature- or pH-stable. Heating above 50 °C leads to denaturation and loss of the sweet taste [19]. Thermal stability might be increased by modifying and mutating the protein [14,15].

Brazzein was isolated from the ripe fruits of the wild West African plant *Pentadiplandra brazzeana* [20]. It is the smallest of the sweet-tasting proteins. The brazzein peptide chain, consisting of 54 amino acids, has a molecular weight of only 6.5 kDa. The structure’s simplicity and stabilization by four disulfide bonds determines brazzein’s high resistance to elevated temperatures and extreme pH values. The protein does not lose its properties during a 2 h incubation at 98 °C and at a 4 h 80 °C incubation in a 2.5–8 pH range [20]. Brazzein is 500–2000 times sweeter than sucrose, depending on the concentration of the comparison solution [20]. The solubility of brazzein is at least 50 mg/mL [21]. Brazzein’s high thermal stability, good solubility, and taste make it one of the most promising candidates for application in the food industry. An additional argument in favor of brazzein is the fact that indigenous people have used it for centuries as a sweetener for drinks and food [22]. Though some parts of sweet-tasting protein polypeptide chains share similarities with known allergens [23], in vitro studies have shown the high antioxidant, anti-inflammatory, and anti-allergenic activities of brazzein [24].

The official approval and introduction of new food additives and ingredients into the food industry requires detailed studies of possible negative effects. So far, only recombinant thaumatin is officially permitted as both a sweetener and taste enhancer in the United States and in the European Union [12]. The safety of thaumatin was confirmed by studies conducted in 1986. The protein was found not to have any general toxic, mutagenic, or teratogenic effects [25]. This article presents the results of our study of the safety of two recombinant sweet-tasting proteins—brazzein and monellin—performed on three animal species (mice, rats, guinea pigs) regarding their acute, subchronic, chronic toxicity, and allergenic and mutagenic properties.

## 2. Materials and Methods

### 2.1. Preparation of Brazzein and Monellin

Recombinant brazzein and monellin were designed and provided by the Shemyakin-Ovchinnikov Institute of Bioorganic Chemistry, Moscow, Russia. In brief, the recombinant brazzein sequence used in this study was identical to the natural one: DKCKKVYENYPVSKCQLANQCNYDCKLDKHARSGECFYDEKRNLQCICDYCEY. Recombinant single chain monellin was constructed by fusing the natural monellin chain B with chain A via a Gly-Phe linker. The sequence of the single-chain monellin obtained is represented below: GEWEIIDIGPFTQNLGKFAVDEENKIGQYGRLTFNKVIRPCMKKTIYEENGFREIKGYEYQLYVYASDKLFRADISEDYKTRGRKLLRFNGPVPPP. Both recombinant proteins were produced in *Pichia pastoris* yeast [26]. The proteins were purified using chromatography followed by 2 steps of ultrafiltration and lyophilization. The identification of proteins was performed using expression construct sequencing followed by both PAA gel electrophoresis and mass-spectrometry. The microbiological purity of sweet proteins was evaluated using bacteriological methods.

### 2.2. Acute Toxicity

Acute toxicity was studied in two species of laboratory animals: mature outbred rats aged 3–4 months with an average body weight of 180–210 g, and mature outbred mice aged 2–2.5 months with an average body weight of 18–21 g. The animals were obtained from a nursery operating according to GLP standards, were bred especially for this study and were not involved in any experiments before. The animals were kept in controlled conditions: at an air temperature of 20–22 °C and a relative humidity of 60–70%. For each experiment, the animals were divided into three groups: one experimental and two control ones. The distribution of animals into groups was carried out randomly, using body weight as a criterion. Individual values of body weight did not deviate from the average value in the group by more than 10%. Each group of rats consisted of 6 animals. Each group of mice consisted of 10 animals. Animals of the experimental group were administered with the tested proteins; animals of the control groups were administered with distilled water or sucrose solution used as a comparative substance. Sucrose (Dia-M, Moscow, Russia) was administered intragastrically to animals of the control group at an equivalent dose (ED), calculated based on the daily norm for humans of 714 mg/kg body weight. The sucrose ED for rat (ED_rat_) and mouse (ED_mouse_) was 4284 mg/kg of body weight and 9282 mg/kg of body weight, respectively. Brazzein has a sweetness 2000-fold higher than sucrose; therefore, ED_rat_ and ED_mouse_ for brazzein were 2.14 mg/kg and 4.64 mg/kg of body weight, respectively. Monellin has a sweetness 3000 times higher compared to sucrose; therefore, ED_rat_ and ED_mouse_ for monellin are 1.43 mg/kg of body weight, and 3.10 mg/kg of body weight, respectively. The tested brazzein and monellin were administered intragastrically once in the form of an aqueous solution at various dose levels, which make it possible to calculate the values of LD50 (LD1; LD16; LD84 and LD99) or to determine the maximum tolerated doses. Two series of experiments were conducted for each protein.

In the first series of experiments, brazzein was administered to rats at dose levels of 107 mg/kg of body weight (50 ED_rat_), 214 mg/kg of body weight (100 ED_rat_), and 1070 mg/kg of body weight (500 ED_rat_); it was administered to mice at dose levels of 232 mg/kg of body weight (50 ED_mouse_), 464 mg/kg of body weight (100 ED_mouse_), and 2320 mg/kg of body weight (500 ED_mouse_).

In the first series of experiments, monellin was administered to rats at dose levels of 71 mg/kg of body weight (50 ED_rat_), 143 mg/kg of body weight (100 ED_rat_), and 715 mg/kg of body weight (500 ED_rat_); it was administered to mice at dose levels of 155 mg/kg of body weight (50 ED_mouse_), 310 mg/kg of body weight (100 ED_mouse_), and 1550 mg/kg of body weight (500 ED_mouse_).

In the second series of experiments, brazzein was administered to rats at dose levels of 1000 mg/kg of body weight (467 ED_rat_), 2500 mg/kg of body weight (1170 ED_rat_), and 5000 mg/kg of body weight (2350 ED_rat_); it was administered to mice at dose levels of 1000 mg/kg of body weight (216 ED_mouse_), 2500 mg/kg of body weight (540 ED_mouse_), and 5000 mg/kg of body weight (1080 ED_mouse_).

In the second series of experiments, monellin was administered to rats at dose levels of 1000 mg/kg of body weight (700 ED_rat_), 2500 mg/kg of body weight (1750 ED_rat_), and 5000 mg/kg of body weight (3500 ED_rat_); it was administered to mice at dose levels of 1000 mg/kg of body weight (320 ED_mouse_), 2500 mg/kg of body weight (800 ED_mouse_), and 5000 mg/kg of body weight (1600 ED_mouse_).

Following agent administration, the animals were kept under continuous observation for the first 30 min, then hourly for the period of 4 h, then after 24 h. Then, the observations were carried out once a day. The duration of observations was 14 days. The criteria for assessing acute toxicity were the death of experimental animals and the timing of their death, the clinical symptoms of intoxication, behavioral reactions, and macroscopic analysis of the organs of fallen and euthanized animals at the end of the experiment. Behavioral reactions were evaluated by visual observations of motor activity, reactions to external stimuli and the setting of behavioral tests in the “open field” test (stand without support, stand with support, short and long grooming, the number of crossed squares, mink effect, exit to the center and defecation). Fourteen days after the administration of the agents, rats and mice from each experimental and control group were euthanized for further pathomorphological studies of internal organs. During necropsy, the external state of the body, the thoracic, abdominal and pelvic cavities with organs and tissues located in them were examined. The following internal organs were subjected to macroscopic examination for visible pathological changes: liver, lungs, kidneys, heart, spleen, brain, pancreas, esophagus, stomach, small intestine, testes (in males) and ovaries (in females).

### 2.3. Subchronic Toxicity

Subchronic toxicity study was carried out on guinea pigs aged 2–2.5 months with an average body weight of 250–300 g. The principles and standards of the keeping, selection, and preparation of animals for the experiment were like those described for the study of acute toxicity. For each experiment, the animals were divided into three groups: two experimental ones and one control. Each group consisted of 9 animals. Animals in the experimental groups were administrated intragastrically with aqueous solutions of brazzein or monellin; animals from the control group were administrated intragastrically with distilled water. The equivalent dose of sucrose (ED) for guinea pigs was 4350 mg/kg body weight. ED_guinea pig,_ calculated accounting the sweetness coefficient for brazzein and monellin, were 2.17 mg/kg of body weight and 1.45 mg/kg of body weight, respectively. Animals from the first experimental group were administrated with the tested proteins at a dose equivalent to 1 ED_guinea pig_, and from the second experimental group at a dose equivalent to 10 ED_guinea pig_. Protein solutions and water were administered daily at the same time for 21 days.

The behavior of guinea pigs (as described in Section 2.2), visible physiological functions, symptoms of intoxication, and possible death of animals were monitored during the entire period of the experiment. The body weight of guinea pigs was recorded daily. Weight gains relative to the initial body weight were determined at the end of the experiment. A biochemical blood test for 16 parameters was used as a criterion for assessing subchronic toxicity. The following parameters were determined: total and direct bilirubin, the activity of aspartate aminotransferase, alanine aminotransferase and pancreatic amylase, the Ritis coefficient, the concentration of urea and creatinine, the content of total protein and glucose, the activity of alkaline phosphatase, lactate dehydrogenase and total alpha-amylase, and the content of fructosamine, triglycerides and cholesterol. Biochemical blood parameters were determined using analyzer Clima MC-15 (RAL Tecnica para el Laboratorio S.A., Barcelona, Spain).

### 2.4. Chronic Toxicity

Chronic toxicity was studied on outbred rats aged 3–4 months with an average body weight of 180–210 g. The principles and standards of the keeping, selection, and preparation of animals for the experiment were like described for the acute toxicity study. Animals were divided into four groups for each experiment: two experimental ones and two control ones. Each group consisted of 10 animals. Animals in the experimental groups were administered intragastrically with aqueous solutions of brazzein or monellin; animals from the control groups were administered intragastrically with an aqueous solution of sucrose or distilled water. Animals from the first experimental group were administered with the tested proteins at a dose equivalent to 1 ED_rat_ (2.14 mg/kg of body weight and 1.43 mg/kg of body weight for brazzein and monellin, respectively), and from the second experimental group at a dose level equivalent to 10 ED_rat_ (21.4 mg/kg of body weight and 14.3 mg/kg of body weight for brazzein and monellin, respectively). Proteins and control solutions were administered daily at the same time for 150 days.

Behavior of rats, visible physiological functions, symptoms of intoxication, and possible death of animals were monitored during the entire period of experiment. The body weight of guinea pigs was recorded daily. Weight gains relative to the initial body weight were determined at the end of the experiment.

The functional state of the central nervous system was assessed by visual observations of motor activity and behavioral reactions in the “open field” test as described in Section 2.2.

The functional state of the kidneys was assessed by diuresis values for 3 h with a water load in rats with a similar body weight and by clinical urine analysis using ATAGO SUR-NE refractometer (ATAGO Co., Ltd., Tokyo, Japan) and URS-20 test strips (ATAGO Co., Ltd., Tokyo, Japan ).

After performing vital tests, animals from each group were euthanized and blood samples (with and without anticoagulant) were taken to determine hematological and biochemical parameters. The main parameters of the peripheral blood (hematocrit, hemoglobin level, number of erythrocytes, leukocytes, platelets, reticulocytes, average hemoglobin content in erythrocytes, average hemoglobin concentration in erythrocytes, average volume of erythrocytes, and erythrocyte anisocytosis index) were determined using hematological analyzer MicroCC-20 Plus (High Technology, Inc., North Attleborough, MA, USA). Biochemical blood parameters (17 parameters: the same as for subchronic toxicity plus insulin) were determined using analyzer Clima MC-15 (RAL Tecnica para el Laboratorio S.A., Barcelona, Spain).

Internal organ collection was obtained after euthanasia for macroscopic examination (as described in Section 2.2) and preparation of microscopic preparations for further histological examination. Collected material was fixed in 10% formalin and filled with paraffin. Histological sections were made on the microtome “Microm HM325” (MICROM International GmbH, Walldorf, Germany) and stained with hematoxylin–eosin. Microscopic preparations were examined using microscope Levenhuk 625 (OJSC Levenhuk, St-Petersburg, Russia).

### 2.5. Allergenic Effects

Allergenic effects were studied on two species of laboratory animals: albino males of guinea pigs aged 3–4 months with an average body weight of 250–300 g, and males of outbred mice aged 2–2.5 months with an average body weight of 18–20 g. The principles and standards of keeping, selection, and preparation of animals for the experiment were like those in the study of acute toxicity.

Guinea pigs, which have a high sensitivity to allergens of different origin, were used to perform skin, conjunctival and nasal tests of brazzein and monellin for allergenic action, as well as for testing the indirect mast cell degranulation reaction [27]. Animals were divided into three groups for each experiment: two experimental ones and one control one. Each group consisted of 12 animals. Animals in the experimental groups were administered intragastrically with aqueous solutions of brazzein or monellin; animals from the control group were administered intragastrically with distilled water. Animals from the first experimental group were administered with the tested proteins at the dose level equivalent to 1 ED_guinea pig_ (2.17 mg/kg of body weight and 1.45 mg/kg of body weight for brazzein and monellin, respectively), and from the second experimental group at a dose level equivalent to 10 ED_guinea pig_ (21.7 mg/kg of body weight and 14.5 mg/kg of body weight for brazzein and monellin, respectively). Protein solutions and water were administered daily at the same time for 21 days.

Guinea pigs were tested on 10–12 days after sensibilization. For the skin test, a small amount of the test agent was applied to the side trimmed (2 × 2 cm) surface of the animal’s skin. The skin reaction was evaluated on a point scale. For the conjunctival test, a drop of the test agent solution was pipetted into the conjunctival sac; 1 drop of 0.9% sodium chloride solution was administered into the second eye (control). The reaction was considered after 15 min (immediate-type reaction) and after 24–48 h (delayed-type hypersensitivity) and evaluated in points. For a nasal test, a solution of the test preparation was pipetted into the nasal passages of experimental and control animals. The results were evaluated after 10–15 min and after 24–72 h according to the reaction.

Exudate draining from intestinal loops was collected from animals subjected to euthanasia for testing the indirect mast cell degranulation reaction. A suspension of mast cells was treated with blood serum of experimental animals and a solution of the tested protein. Next, the number of degranulated cells per 100 mast cells was calculated under a microscope and the average of 3 repetitions was calculated. 

Mice were used to test the inflammatory response to concanavalin A. Animals were divided into three groups for each experiment: two experimental and one control. Each group consisted of 10 animals. Animals in the experimental groups were administered intragastrically with aqueous solutions of brazzein or monellin, animals from the control group were administered intragastrically with distilled water. Animals from the first experimental group were administered with the studied proteins at a dose level equivalent to 1 ED_mouse_ (4.64 mg/kg of body weight and 3.10 mg/kg of body weight for brazzein and monellin, respectively), and from the second experimental group at a dose level equivalent to 10 ED_mouse_ (46.4 mg/kg of body weight and 31.0 mg/kg of body weight for brazzein and monellin, respectively). Protein solutions and water were administered daily at the same time for 21 days.

Two hours following the last administration of tested proteins, mice of the experimental and control groups were injected subplantarly (into the pad of the hind paw) by concanavalin A (Sigma-Aldrich, St.-Luis, MO, USA) at the dose level of 100 μg/mouse; 0.9% NaCl solution was injected into the opposite paw. One hour after injection, the mice were euthanized, the paws were weighed, and the index of the inflammatory reaction was calculated by the following formula:

$$Ii\left(\%\right)=\frac{Pexp-Pcon}{Pcon}\times 100\%$$

where

*Ii*—inflammatory index;

*Pexp*—weight of the paw injected with concanavalin A;

*Pcon*—weight of the paw injected with 0.9% NaCl solution.

### 2.6. Mutagenic Effects

Experiments in vivo on mutagenic effects of brazzein and monellin were performed on mice using a mammalian bone-marrow chromosomal aberration test and micronucleus test in polychromatophilic bone-marrow erythrocytes, as well as in vitro in an Ames test using an indicator strain of *Salmonella typhimurium* to assess the ability of studying proteins to induce gene mutations.

#### 2.6.1. Ames Test

The Ames test was performed according to the described method [28]. The Ames test uses the strains of the bacterium Salmonella typhimurium that carry mutations in genes involved in histidine synthesis. These strains require histidine for growth, but cannot produce it. The method tests the capability of the tested substance in unducing mutations that result in cells growth in a histidine-free medium. A set of indicator strains of *S. typhimurium* used for the study allows registering mutations such as reading frame shift (TA 98 and TA 97) and base pair substitutions (TA 100). Brazzein or monellin was tested at five concentrations by adding 0.1 mL of prepared solutions to a Petri dish (50,000; 10,000; 2000; 400 and 80 µg/mL). To control the activity of the metabolic activation system, ethidium bromide (10 µg per dish, TA 98 strain) was used. Distilled water (0.1 mL per dish) was used as a negative control. The microsomal fraction S9 of the liver of Wistar rats was used for metabolic activation. The results were recorded post 48 h of incubation.

#### 2.6.2. Mammalian Bone Marrow Chromosomal Aberration Test and Micronucleus Test

Mice F1 (CBAxC57Bl/6) aged 2–2.5 months with average body weight 18–21 g were used to test mutagenic effects on mammals. The principles and standards of the keeping, selection, and preparation of animals for the experiment were like described for the acute toxicity study. Animals were divided into seven groups for each experiment: two negative controls (one male and one female group), one positive control (male group), and four experimental (three male groups and one female group). Each group consisted of 6 animals.

Animals of the first (males) and second (females) negative control groups were administered intragastrically with distilled water, once a day for 4 days with an interval of 24 h.

Animals of the third group of the positive control were injected once intragastrically with endoxane (cyclophosphamide) at a dose of 20 mg/kg of body weight.

Animals of the fourth (males) experimental group were administered once intragastrically with ED_mouse_ of brazzein or monellin (4.64 mg/kg of body weight and 3.10 mg/kg of body weight, respectively).

Animals of the fifth (males) experimental group were administered once with brazzein or monellin at the maximum dose level possible for intragastric administration, determined in the acute toxicity experiment (5000 mg/kg body weight).

Animals of the sixth (males) and seventh (females) experimental groups were administered with ED_mouse_ brazzein or monellin (4.64 mg/kg of body weight and 3.10 mg/kg body weight, respectively) intragastrically, once a day for 4 days with an interval of 24 h.

Preparations of bone marrow cells to account for chromosomal aberrations and micronuclei were prepared in accordance with the described method [29]. The analysis of the preparations was carried out using light microscopy at a magnification of 10 × 100 with oil immersion. To account for chromosomal aberrations, rounded metaphase plates were analyzed without chromosome overlays with a modal number of 39–40, and 100 metaphases from each animal. Single and paired fragments, chromatid and chromosomal exchanges, and cells with multiple chromosomal aberrations were considered. To account for micronuclei, 2000 polychromatophilic erythrocytes (PCE) from each animal were analyzed to account for PCE with micronuclei. The proportion of PCE from the total amount of erythrocytes (the sum of PCE and normochromic erythrocytes) was determined with an additional count of 500 erythrocytes.

### 2.7. Statistics

Statistical processing of the data on the dynamics of body weight gain and mass coefficients was carried out by using a simple comparison of averages according to the Student’s t-criterion. The differences were determined at significance level 0.05.

Statistical processing of the data obtained in the “open field” test was carried out using a set of nonparametric criteria (Mann–Whitney U test, Kruskal—Wallis test, etc.)

Statistical processing of the data on accounting for chromosomal aberrations and micronuclei was carried out separately for males and females by comparing experimental groups with control groups using the Mann–Whitney criterion at a significance level of 0.05 for each of the comparisons.

### 2.8. Regulating Acts and Guidelines

All the above experiments on testing the toxicity, allergenicity and mutagenicity of brazzein and monellin were performed in strict compliance with the international and national regulatory acts and guidelines [30,31,32].

### 2.9. Bioethics

The use of laboratory animals and the planning of the experiments was agreed with the Bioethical Commission of the Scientific Research Centre “Pharmbiomed” and recommended for use. The personnel involved in the experiments was trained in the correct and humane treatment of laboratory animals.

All painful manipulations with animals were carried out in accordance with the regulatory standards [33,34,35].

## 3. Results

**Figure 1:**
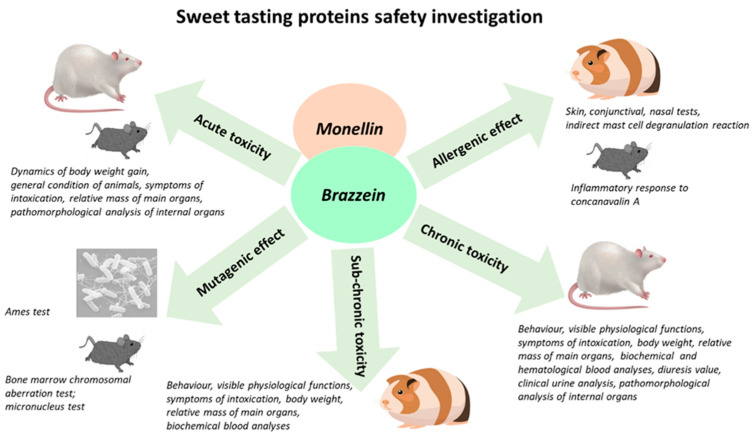
Experiment scheme for assessment of recombinant brazzein and monellin safety.

The scheme of the experiment to assess the safety of brazzein and monellin was developed to obtain the most reliable results using the maximum number of animals and bacterial models (Figure 1).

### 3.1. Acute Toxicity

The acute toxicity experiment on rats (males and females) and mice (males and females) established that both for brazzein and monellin, the LD50 appeared to be over 5000 mg/kg of body weight when administered intragastrically. Symptoms of animal intoxication and death were not observed for any tested dose during the entire experiment duration. The exact value of LD50 could not be established due to the low toxicity of the tested proteins. In the reference and control groups of animals administered with sucrose solution and distilled water, respectively, death and symptoms of intoxication were also not observed. We found no changes in the behavior of experimental animals in the “open field” test compared to the control groups.

Body weight gain dynamics is an additional index reflecting the toxic effects of the tested proteins. Intragastric administration of brazzein or monellin to rat males and females at dose level ranges of 107 mg/kg to 5000 mg/kg and 71 mg/kg to 5000 mg/kg body weight, respectively, did not significantly affect body weight gain dynamics or the mass coefficients of internal organs.

Similar results were obtained in experiments assessing acute toxicity in mice. The intragastric administration of brazzein or monellin to male and female mice at dose level ranges of 232 mg/kg to 5000 mg/kg and 155 mg/kg to 5000 mg/kg body weight, respectively, did not lead to animal death or intoxication symptoms. The administration of brazzein or monellin at all doses did not significantly affect mice weight gain values or internal organ mass coefficients.

The macroscopic study of internal organs after intragastric administration of brazzein or monellin to rats and mice at all tested doses revealed no pathological changes in the liver, lungs, kidneys, heart, spleen, brain, lymph nodes, pancreas, thymus, esophagus, stomach, small intestine, large intestine, testes, or ovaries. There were no pathological changes in the shape of organs and the color of the outer coverings, texture and color of tissues, pathological inclusions and formations.

The maximum tested dose of brazzein or monellin for rats and mice used in this study is 5000 mg/kg body weight, close to the maximum possible dose for intragastric administration to animals of these species. When a dose of 5000 mg/kg of body weight was administered to rats and mice of both sexes, no intoxication symptoms or death occurred. Since LD50 and other toxicological parameters are statistical values, lethal doses are required to calculate them, including an absolutely lethal dose that causes the death of 100% of animals. Since the maximum tolerated, toxic, and lethal doses could not be established, it is not possible to determine the exact LD50 value of brazzein and monellin.

### 3.2. Subchronic Toxicity

In experiments to assess the subchronic toxicity of brazzein and monellin on guinea pigs, lethal cases and any symptoms of intoxication were not observed.

Macroscopic examination and internal organ mass coefficient determination of experimental animals revealed no pathological changes.

During the experiment, no significant deviations of the biochemical parameters of experimental animals from the reference values or from the indicators of the control groups were found.

The absence of guinea pig death or intoxication symptoms, the absence of negative effects on body weight gain dynamics, the internal organ mass coefficients, and the biochemical indices showed that daily intragastric administration of brazzein or monellin for 21 days at doses of ED_guinea pig_ and 10ED_guinea pig_ does not demonstrate any toxic effects on guinea pigs, a species that is extremely sensitive to various exposures.

### 3.3. Chronic Toxicity

To obtain convincing and substantiated conclusions about brazzein and monellin chronic toxicity, methodologically different protocols such as the visual observation of animals, determination of body weight gain dynamics, hematological and biochemical indices, diuresis and urinalysis, and an assessment of rat individual behavior using an «open field» test were used. The experiment culminated with a macroscopic and microscopic organ examination.

The relative weight gain in male rats in experiments to assess the chronic toxicity of brazzein with intragastric administration was reduced. By the end of the experiment period, this indicator for the tested protein administered in doses of ED_rat_ and 10ED_rat_ was 337.94 ± 9.56% and 326.91 ± 14.41%, respectively, with a control value of 389.40 ± 13.73%. In the group of males treated with sucrose, the average weight gain of 341.95 ± 8.16% was also significantly lower than in the control group.

Usually, a decrease in weight gain is associated with toxic effects, but in this experiment, no toxic effects were detected by other indicators. We believe that in this case, the reduction in weight gain is the result of a slight decrease in feed intake because of the administration of brazzein or sucrose, which causes the animals to reach satiety. In particular, the average daily feed intake with the administration of the studied protein was at a dose of ED_rat_ 21.52 ± 0.71 g per capita (t = 4.24), at a dose of 10 ED_rat_ 23.53 ± 0.54 g per capita (t = 2.35); with the administration of sucrose this was 21.25 ± 0.47 g per capita (t = 5.73). The average daily feed intake in control group was 25.33 ± 0.52 g per capita. Judging by the absolute values of body weight, the decrease in feed intake began approximately 1 month after the start of the administration of brazzein and sucrose.

The results of observations of weight gain in female rats in chronic toxicity experiment show that the dynamics of body weight gain after the administration of brazzein did not undergo statistically significant changes. After administration of the studied protein in doses of ED_rat_ and 10ED_rat_, the relative weight gain was 191.25 ± 6.31% and 198.07 ± 9.45%, respectively. In the control group, the relative weight gain was 197.04 ± 7.07%. In female rats treated with sucrose, there was a tendency to decrease weight gain (183.05 ± 5.07%) compared with the control group.

Nevertheless, despite the absence of weight gain reduction, there was also a decrease in feed intake in female rats. The average daily feed intake with the administration of brazzein at doses of ED_rat_ and 10ED_rat_ was 15.54 ± 0.40 g per capita (t = 2.92) and 15.46 ± 0.50 g per capita (t = 2.72), respectively. With the administration of sucrose, it was 15.75 ± 0.41 g per capita (t = 2.54). In the control group of females, the average daily feed intake was 17.20 ± 0.38 g per capita. A decrease in feed intake in female rats, as well as in males, is the result of the development of a feeling of satiety.

Similar results on the dynamics of weight gain were obtained in studies of the chronic toxicity of monellin. The dynamics of body weight gain in male rats also tended to decrease with the administration of the studied protein. By the end of the experiment, the relative weight gain after administration of monellin in doses of ED_rat_ and 10ED_rat_ was 354.44 ± 9.49% and 356.13 ± 10.33%, respectively. In the control group, the relative weight gain was 389.40 ± 13.73%. In the group of male rats treated with sucrose, the body weight gain (341.95 ± 8.16%) was significantly lower compared to the control group. The dynamics of body weight gain in female rats after the period of monellin administration did not undergo statistically significant changes, although there was a downward trend. After administration of monellin at doses of ED_rat_ and 10ED_rat_, the relative weight gain was 182.16 ± 4.35% and 179.56 ± 3.72%, respectively, with a value in the control group of 197.04 ± 7.07%.

The mass coefficients of all studied internal organs of rats, both males and females, treated with brazzein or monellin did not undergo statistically significant changes.

The administration of the studied proteins to male rats at doses of ED_rat_ and 10ED_rat_ did not lead to a significant change in the overall parameters of peripheral blood. The administration of sucrose led to a slight but statistically significant change in the average hemoglobin content in the erythrocyte (18.58 ± 0.21 pg compared with the control value of 19.39 ± 0.18 pg); the average volume of the erythrocyte (58.61 ± 0.46 µm^3^ compared with the control value of 60.79 ± 0.42 µm^3^) and the indicator of erythrocyte anisocytosis (28.41 ± 0.29% compared to with a reference value of 27.05 ± 0.30%). The comprehensive assessment of the chronic toxicity of brazzein and monellin also included the determination of biochemical parameters of blood serum (21 indicators) of rats. After the administration of brazzein at a dose of 10ED_rat_, a decrease in creatinine levels was noted (72.30 ± 2.58 µmol/L compared with the control value of 81.70 ± 2.72 µmol/L), which led to a change in the urea/creatinine ratio.

The administration of brazzein caused a slight but significant decrease in the level of total protein and globulin in male rats, as well as an increase in glucose concentration compared with a low control value.

There was a decrease in the level of fructosamine (165.80 ± 16.58 µmol/L compared with the control value of 225.30 ± 7.07 µmol/L) in the blood serum of female rats after administration of brazzein at a dose of ED_rat_, and an increase in the level of triglycerides when administered at a dose of 10ED_rat_ (2.83 ± 0.26 µmol/L versus 1.96 ± 0.19 µmol/L in the control). The introduction of the studied protein in a dose of ED_rat_ also led to a decrease in LDG activity. A dose of 10ED_rat_ did not have such an effect.

The administration of sucrose to female rats caused an increase in the level of urea (7.91 ± 0.44 mmol/L compared with the control value of 6.43 ± 0.33 mmol/L), as a result of which the calculated ratio of urea/creatinine changed.

Monellin, when administered at a dose of ED_rat_, also led to a slight but significant decrease in the level of total protein and albumin in the blood of male rats, but this effect was absent when the studied protein was administered at a dose of 10ED_rat_. The administration of monellin at a dose of 10ED_rat_ caused an increase in glucose concentration compared to a low control value.

In the blood serum of female rats, when monellin was administered at a dose of ED_rat_, there was a decrease in the level of fructosamine (164.40 ± 17.93 µmol/L compared with the control value of 225.30 ± 7.07 µmol/L) and cholesterol concentration (1.69 ± 0.06 mmol/L versus 2.23 ± 0.10 mmol/L in the control). When the test protein was administered at a higher dose of 10ED_rat_, there were no such deviations.

As a result of these studies, certain changes in the biochemical parameters of the serum of male and female rats were noted after the administration of brazzein and monellin. Because almost all the changed indicators fall within the range of reference values for this animal species, as well as the absence of a noticeable tendency to increase or decrease in indicators and the absence of a clear dose–effect relationship, all the above-mentioned changes in blood parameters can be interpreted as insignificant and reflecting fluctuations within the physiological norm.

The administration of brazzein or monellin in all tested doses had no statistically significant effect on behavioral reactions in any cases, such as a stand without support, a stand with support, short and prolonged grooming, the number of crossed squares, the mink effect, going to the center and defecation. The administration of sucrose also did not affect the indicators of individual behavior of rats. During the administration of the studied proteins, the rats retained motor activity and responses comparable to the period before the start of the experiment and those observed in control animals.

A day after the last administration of brazzein at doses of ED_rat_ and 10ED_rat_, the indicators of excretory kidney function (daily diuresis) in male rats were 15.40 ± 1.21 mL (statistically significant increase) and 14.40 ± 0.98 mL, respectively, compared with the control value of 10.70 ± 1.25 mL. After the administration of sucrose, the daily diuresis was 10.50 ± 0.50 mL. Diuresis in female rats of all experimental groups after administration of brazzein and sucrose compared with the control group did not change significantly.

The daily diuresis in male rats a day after the last administration of monellin at doses of ED_rat_ and 10ED_rat_ was 15.70 ± 0.86 mL (statistically significant increase) and 11.50 ± 1.87 mL, respectively, compared with the control value of 10.70 ± 1.25 mL. After the introduction of sucrose, the daily diuresis was 10.50 ± 0.50 mL. Diuresis in female rats of all experimental groups after administration of monellin and sucrose compared with the control group did not statistically significantly change.

Kidney function was also evaluated with a clinical analysis of rat urine after administration of the studied proteins. The urine analysis data of experimental rats according to the results of quantitative and qualitative tests practically did not differ from the control values, except for a slight increase in the pH value of urine.

Macroscopic and microscopic examinations of the liver, kidneys, lungs, heart, spleen, pancreas, lymph nodes (mesenteric), stomach, small and large intestines, brain, adrenal glands, thymus, testes (in males), and ovaries (in females) following administration of brazzein, monellin, and sucrose to rats at two tested dose levels did not reveal any pathological changes.

Judging by the external signs, the reactions and behavior of animals, brazzein and monellin did not have an irritating effect on the gastrointestinal mucous, and the rats quickly calmed down after administration of the studied proteins. This observation was confirmed by the results of histological examination of the gastrointestinal mucous, which indicated the absence of pathological changes after prolonged intragastric administration of brazzein and monellin.

### 3.4. Allergenic Effects

#### 3.4.1. Skin Test

Each tested protein was applied to trimmed (hairless) skin areas of guinea pigs on day 10 (the sensitization period) after the last administration of brazzein or monellin. Visual examination after 15 and 30 min and 24 and 48 h revealed no immediate or delayed skin hypersensitivity reactions. Reactions were evaluated on a point scale where 0 points indicates no visible reaction.

In terms of skin color (absence of hyperemia) and skin fold thickness (absence of infiltration), the test areas in control animals did not differ from those in experimental animals.

#### 3.4.2. Conjunctival Test

The reaction of experimental guinea pigs to the conjunctival test did not differ from that of control animals. After receiving a solution of brazzein or monellin, animals first closed their eyes (a natural reaction to foreign matter entering the eyes). There were no changes in the vascular pattern of the eye conjunctiva and no general hyperemia after 15 and 30 min and 24 h. The reaction was assessed as negative.

#### 3.4.3. Nasal Test

Administration of a solution of brazzein or monellin into the nasal passages of guinea pigs did not cause mucosal hyperemia, sneezing, edema, or increased mucus secretion after 15 or 30 min or 24 h, so the test was evaluated as negative.

#### 3.4.4. Indirect Mast Cell Degranulation Reaction

A study of guinea pig blood sera obtained on day 10 after the last administration of brazzein or monellin in the tested doses showed no sensitization to the studied proteins, as the degranulated mast cell percentage in experimental animals did not significantly differ from that of control animals. The results of the indirect reaction test of mast cell degranulation are given in Table 1 and Table 2.

The administration of brazzein and monellin did not lead to any significant change in indirect mast cell degranulation reaction values compared to the control, which indicates that the animal organism is not sensitized to the studied proteins.

#### 3.4.5. Inflammatory Response to Concanavalin A

After brazzein and monellin administration, the index of inflammatory response to concanavalin A in experimental group mice did not significantly differ from that of control animals, which shows the studied proteins have no allergenic activity. The results of the inflammatory response study to concanavalin A are presented in Table 3 and Table 4.

The combined results of skin, nasal, and conjunctival tests, the indirect reaction of mast cell degranulation in guinea pigs, and the inflammatory response to concanavalin A in mice indicate the absence of brazzein and monellin’s allergenic properties.

## 4. Mutagenic Action In Vitro and In Vivo

### 4.1. Ames Test In Vitro

The Ames test results show that the number of revertant colonies on dishes with solvent (negative control) in the MAS- (without metabolic activation system) and MAS+ (with metabolic activation system) variants was within the range of spontaneous level fluctuations for the *S. typhimurium* strains used.

The mutagenic effect, in accordance with generally accepted approaches, is considered significant if the average number of revertant colonies per dish in the experimental version exceeds that of the control by two times or more. The Ames test results assessing the mutagenic activity of brazzein and monellin in vitro are presented in Table 5 and Table 6.

The results presented in Table 5 and Table 6 indicate that brazzein and monellin in all tested concentrations do not have a mutagenic effect in vitro on *S. typhimurium* strains TA 100, TA 98, and TA 97 both in the presence and without a metabolic activation system.

### 4.2. Tests In Vivo

Analysis of the frequency of chromosomal aberrations and the mitotic index in mice bone marrow cells after brazzein administration in each animal and group of animals shows that the control group (oral administration of distilled water) frequency of chromosomal aberrations at metaphase plates was 1.83% for males and 1.00% for females, which coincides with our accumulated laboratory control—no more than 2%. The high frequency of metaphases with chromosomal aberrations detected in the male positive control group administered with Endoxane at a dose level of 20 mg/kg was 14.5 times greater (26.5%) than in the negative control group.

The frequency of metaphases with chromosome aberrations in groups of mice administered with brazzein was 2.33%, 3.17%, 2.17%, and 0.40%, which does not statistically differ from the corresponding negative control groups. Single fragments pre-dominated among the types of aberrations. Select metaphases had paired fragments or exchanges. The mitotic index in the experimental groups varied between 2.33–3.41%, which is within the historical control data for this index.

Analysis of the frequency of polychromatophilic erythrocytes (PCE) with micro-nuclei (MN) and the PCE proportion of the total PCE and normochromic erythrocyte amount in bone marrow in the brazzein experiments, performed for each animal and group of animals, demonstrates that the PCE with micronuclei frequency in the control (oral administration of distilled water) was 1.33% in males and 1.50% in females. This coincides with the accumulated laboratory control—no more than 2%.

Comparing groups of brazzein-treated male mice (groups 4–6) and the male control group (group 1), as well as female groups 2 and 7, there are no statistically significant differences in the frequency of PCE with micronuclei. Endoxane (positive control) at a dose level of 20 mg/kg induced 9.50% PCE with micronuclei, which was seven times greater than in the negative control and was statistically significantly higher than in the groups of brazzein-treated mice.

In the experiment assessing monellin’s mutagenic effects, the frequency of meta-phases with chromosomal aberrations in the control groups (administration of distilled water) was 2.67% for males and 1.83% for females. The high frequency of metaphases with chromosomal aberrations detected in the male positive control group administered with Endoxane at a dose level of 20 mg/kg was 5.3 times greater (14.17%) than in the negative control group.

In the groups of mice that received monellin, the frequency of metaphases with chromosome aberrations was 1.50%, 0.50%, 1.00%, and 0%, which did not statistically exceed the levels of the corresponding negative control. Single fragments predominated among the types of aberrations. Select metaphases had paired fragments or exchanges.

There was a statistically significant increase in the proliferative activity of bone marrow cells under the action of this protein. In the control, the mitotic index was 2.37% for males and 3% for females, whereas the mitotic index in males after a single monellin administration was 5.60%, 11.13% after four administrations, and 11.03% after a single exposure to a dose of 5000 mg/kg, which was 2.4 or 4 times greater than in the control. A similar effect was observed in females who received monellin for 4 days. In this experimental group, the mitotic index was 3.8 times higher than that of the control group.

Analysis of the frequencies of PCE with micronuclei and the PCE proportion in the total PCE and normochromic erythrocyte amount in bone marrow in the monellin experiments, performed for each animal and group of animals, showed that the frequency of PCE with micronuclei in control group animals (oral administration of distilled water) was 1.25% in males and 0.83 in females.

There were no statistically significant differences in the frequency of PCE with micronuclei between male mice groups treated with monellin (groups 4–6) and the male control group (group 1), as well as between female groups 2 and 7 (1.08%, 0.83%, 2.00% and 1.08%). Endoxane (positive control) at a dose of 20 mg/kg induced 4.0% PCE with micronuclei, which was 3.2 times higher than in the negative control group and was statistically significantly higher than in groups of mice treated with monellin at all administration regimes.

The bone marrow chromosomal aberration test showed that brazzein and monellin had no cytogenetic activity in mouse bone marrow cells when administered intragastrically in the above doses. Additionally, the micronucleus test showed that the studied proteins, in the tested doses, had no cytogenetic activity in mice bone marrow polychromatophilic erythrocytes.

A battery of short-term screening tests is widely used to predict carcinogenic activity, with the main ones being the Ames test, the chromosome aberration test, and the micronucleus test. The negative results of all three tests in our case indicate the absence of mutagenic properties, which also gives grounds to predict the absence of carcinogenic properties.

## 5. Discussion

In addition to the obvious advantages that sweet-tasting proteins hold over sugar in that they do not cause obesity risk and do not contribute to dental problems, they could be used in food at much lower concentrations. Moreover, the gene sequence coding for sweet proteins can be easily modified to achieve optimal sweetness and can be expressed at high levels in heterologous hosts such as bacteria, yeast, and plants [36]. Despite the long history of studying sweet proteins and the intensive studies of their structure and recognition mechanism by taste receptors, only thaumatin has been approved for commercial use as a sweetener and flavor enhancer [37]. Perhaps this is due to the lack or small number of studies devoted to assessing the safety of other sweet proteins.

We performed a wide and detailed safety study of brazzein and monellin, two promising protein sweeteners, which could become sugar substitutes or food ingredients. The potential for brazzein as a heat-stable, water-soluble, nutritive sweetener is well recognized. Monellin has a more complex structure and is more sensitive to heat, though its modifications were reported to be more stable [26]. Recombinant brazzein and monellin expression has been achieved on several platforms, with the goal of making a low-cost, commercializable version [36]. Despite numerous published studies of the structure of these sweet proteins and reports of various obtained recombinant brazzein and monellin modifications in several producing organisms, a systematic study of their safety, to the best of our knowledge, has not been conducted.

Rodents are standard animal models for testing the toxicity of biologically active substances. Rats and mice are recommended in various regulatory documents as one of the most adequate test systems for the general toxicity evaluation of biologically active substances with any mode of action. In our study, a third animal species—guinea pigs—was added in some experiments, as they are often more sensitive to external influences and toxicants than rats and mice.

In the acute toxicity experiment, we observed no signs of toxic effects from both proteins given at single doses as high as 5000 mg/kg of body weight in the animal models used. In the chronic toxicity experiments, no toxic effects were recorded as well, evidencing the absence of a functional cumulative effect. The deviations identified in some biochemical parameters are few and most of them are results of indices outliers in individual animals, and the changed indices themselves are within the reference value range for their animal species.

It should be emphasized that experiment was carried out in rather “stringent” conditions: in addition to the equivalent dose (ED_rat_), a tenfold equivalent dose (10ED_rat_) was used. Additionally, the administration period was as long as 150 days. As a result, the total doses received by rats of both genders at single daily dose regime of 2.14 mg/kg (ED_rat_) and at single daily dose regime of 21.4 mg/kg (10ED_rat_) were 321 mg and 3210 mg, respectively.

As the exposure progressed, i.e., with an increase in the number of administrations, the toxic effects of the tested agents were not recorded, which evidences the absence of a functional cumulative effect.

Developing and integrating new food products, including food ingredients, into the daily human diet necessitates studies of their possible sensitizing properties. Testing the allergenic effects of brazzein and monellin is important considering the proteinaceous nature of these agents and their prospects for wide application as food ingredients. Based on the results of the performed studies, it can be concluded that brazzein and monellin do not demonstrate allergenic effects. An equally important property that candidates for dietary supplements should have is the absence of mutagenic and cytogenetic effects. The results of the present study convincingly show that brazzein and monellin do not induce gene mutations in the Ames test on *S. typhimurium* and cytogenetic activity in mouse bone marrow cells.

Further studies on reproductive toxicity and toxicity on immature animals are required to answer the question whether monellin and brazzein could be potentially safe for consumption by children and pregnant woman.

Also the studies of the fecal microbiota and animal metabolome to obtain data on the effect of sweet proteins on the composition of the microbiota and their effect on metabo-lism would be of interest.

Our study of brazzein and monellin safety, performed using a wide range of model systems and methods, perhaps for the first time since the introduction of thaumatin opens promising prospects for the use of these proteins as sugar substitutes.

## 6. Conclusions

Animal appearance and behavior, the absence of any clinical signs of intoxication, the lack of mortality, the body weight gain dynamics, the organ mass coefficients, the hematological and biochemical indices, the functional states of the liver, kidneys, and central nervous system, the morphology and histology of multiple organs, and the absence of local irritating effects on gut mucous membranes evidenced that brazzein and monellin appear to be highly safe in mammal experimental models.

The results of skin, nasal, and conjunctival tests, the indirect reaction of mast cell degranulation in guinea pigs, and the inflammation reaction to concanavalin A in mice indicate that brazzein and monellin proteins have no allergenic properties.

The absence of induced chromosomal aberrations in mice bone marrow cells for different administration regimes and doses, the absence of micronuclei accumulation in the polychromatophilic erythrocytes of mice bone marrow cell populations, and the negative Ames test allow us to conclude that brazzein and monellin do not demonstrate cytogenetic and mutagenic activities.

## Figures and Tables

**Table 1:** Test of indirect reaction of mast cell degranulation with guinea pig blood sera after sensitization with brazzein in two doses (*n* = 6).

Administration	Dose	Result (%) (M ± m)
Brazzein	ED_guinea pig_	5.56 ± 0.19 (t = 1.51)
Brazzein	10ED_guinea pig_	5.78 ± 0.22 (t = 2.11)
Distilled water (control)	0.5 mL per capita	5.17 ± 0.14

In all cases *p* ≥ 0.05 (t_crit_ = 2.23).

**Table 2:** Test of indirect reaction of mast cell degranulation with guinea pig blood sera after sensitization with monellin in two doses (*n* = 6).

Administration	Dose	Result (%) (M ± m)
Monellin	ED_guinea pig_	5.50 ± 0.14 (t = 1.51)
Monellin	10ED_guinea pig_	5.72 ± 0.22 (t = 1.94)
Distilled water (control)	0.5 mL per capita	5.17 ± 0.14

In all cases *p* ≥ 0.05 (t_crit_ = 2.23).

**Table 3:** Results of the inflammatory response to concanavalin A in mice after administration of brazzein in two doses (*n* = 10).

Administration	Dose	Result (%) (M ± m)
Brazzein	ED_guinea pig_	17.98 ± 1.53 (t = 0.75)
Brazzein	10ED_guinea pig_	18.07 ± 1.01 (t = 0.91)
Distilled water (control)	0.02 mL per capita	16.20 ± 1.64

In all cases *p* ≥ 0.05 (t_crit_ = 2.10).

**Table 4:** Results of the inflammatory response to concanavalin A in mice after administration of monellin in two doses (*n* = 10).

Administration	Dose	Result (%) (M ± m)
Monellin	ED_guinea pig_	17.33 ± 1.81 (t = 0.44)
Monellin	10ED_guinea pig_	17.48 ± 1.75 (t = 0.50)
Distilled water (control)	0.02 mL per capita	16.20 ± 1.64

In all cases *p* ≥ 0.05 (t_crit_ = 2.10).

**Table 5:** Results of mutagenic activity assessment of brazzein in the Ames test.

Strain	Metabolic Activation	Agent Concentration, µg/mL	Number of Revertant Colonies per Dish				X_avexp_/X_avcon_
			X_1_	X_2_	X_3_	X_av_	
TA 100	MAS-	50,000	115	104	108	109.0	0.91
		10,000	107	119	102	109.3	0.92
		2000	114	110	116	113.3	0.95
		400	121	115	111	115.7	0.97
		80	119	113	109	113.7	0.96
		H_2_O	122	108	126	118.7	
		Sodium azide	1174	1352	1430	1318.7	11.11 *
	MAS+	50,000	102	91	114	102.3	0.92
		10,000	100	116	110	108.7	0.98
		2000	112	94	104	103.3	0.93
		400	114	107	121	114.0	1.02
		80	123	111	115	116.3	1.05
		H_2_O	116	120	98	111.3	
TA 98	MAS-	50,000	16	23	24	21.0	0.89
		10,000	24	26	21	23.7	1.00
		2000	30	25	22	25.7	1.08
		400	26	27	19	24.0	1.01
		80	21	26	20	22.3	0.94
		H_2_O	23	21	27	23.7	
		DMSO	22	28	30	26.7	
		2NF	148	137	128	137.7	5.16 *
	MAS+	50,000	19	23	21	21.0	0.71
		10,000	26	21	29	24.7	0.83
		2000	27	23	26	25.3	0.85
		400	31	27	22	26.7	0.90
		80	18	25	28	23.7	0.80
		H_2_O	24	32	33	29.7	
		Ethidium bromide	226	247	192	221.7	7.46 *
TA 97	MAS-	50,000	104	106	97	102.3	0.78
		10,000	113	121	128	120.7	0.92
		2000	124	123	132	126.3	0.96
		400	123	120	136	126.3	0.96
		80	139	118	134	130.3	0.99
		H_2_O	135	137	123	131.7	
		DMSO	122	128	134	128.0	
		9AA	592	638	614	614.7	4.80 *
	MAS+	50,000	127	144	129	133.3	0.99
		10,000	135	148	125	136.0	1.01
		2000	125	131	124	126.7	0.95
		400	133	129	130	130.7	0.98
		80	144	123	136	134.3	1.00
		H_2_O	128	142	132	134.0	

Notes: X_1_, X_2_ and X_3_—number of revertant colonies in 1st, 2nd, and 3rd dish; X_av_—average number of colonies per dish; X_avexp_/X_avcon_—ratio of average number of revertant colonies per dish in experiment to that in control; *—significant mutagenic effect (sodium azide, 2NF, 9AA and ethidium bromide—standard mutagens (positive control)).

**Table 6:** Mutagenic activity assessment of monellin in Ames test.

Strain	Metabolic Activation	Agent Concentration, µg/mL	Number of Revertant Colonies per Dish				X_avexp_/X_avcon_
			X_1_	X_2_	X_3_	X_av_	
TA 100	MAS-	50,000	128	124	118	123.3	0.99
		10,000	125	122	113	120.0	0.97
		2000	117	111	125	117.7	0.95
		400	123	117	129	123.0	0.99
		80	114	124	119	119.0	0.96
		H_2_O	125	120	127	124.0	
		Sodium azide	1270	1424	1312	1335.3	10.76 *
	MAS+	50,000	121	123	109	117.7	1.00
		10,000	120	118	124	120.7	1.03
		2000	108	127	106	113.7	0.97
		400	123	119	112	118.0	1.01
		80	126	110	114	116.7	0.99
		H_2_O	119	111	122	117.3	
TA 98	MAS -	50,000	27	25	33	28.3	1.02
		10,000	32	28	20	26.7	0.96
		2000	30	31	23	28.0	1.01
		400	33	27	21	27.0	0.97
		80	24	24	29	25.7	0.93
		H_2_O	24	29	30	27.7	
		DMSO	28	34	26	29.3	
		2NF	132	145	151	142.7	4.87 *
	MAS+	50,000	30	27	25	27.3	0.87
		10,000	34	24	32	30.0	0.96
		2000	34	27	32	31.0	0.99
		400	25	23	37	28.3	0.91
		80	24	33	29	28.0	0.89
		H_2_O	27	36	31	31.3	
		Ethidium bromide	348	272	315	311.7	9.96 *
TA 97	MAS-	50,000	119	129	124	124.0	0.91
		10,000	122	119	125	122.0	0.89
		2000	113	120	126	119.7	0.87
		400	128	124	118	123.3	0.90
		80	125	130	123	126.0	0.92
		H_2_O	133	147	131	137.0	
		DMSO	142	129	132	134.3	
		9AA	713	698	647	686.0	5.01 *
	MAS+	50,000	122	135	120	125.7	0.99
		10,000	133	125	123	127.0	1.00
		2000	124	116	117	119.0	0.94
		400	112	124	126	120.7	0.95
		80	127	121	129	125.7	0.99
		H_2_O	130	123	127	126.7	

Notes: X_1_, X_2_ and X_3_—number of revertant colonies in 1st, 2nd, and 3rd dish; X_av_—average number of colonies per dish; X_avexp_/X_avcon_—ratio of average number of revertant colonies per dish in experiment to that in control; *—significant mutagenic effect (sodium azide, 2NF, 9AA and ethidium bromide—standard mutagens (positive control)).

## Data Availability

All data in this study are available from the corresponding author upon request.
